# ﻿Morphological and multi-locus phylogenetic analyses reveal three new branched species of *Clavaria* (Clavariaceae, Agaricales) from China

**DOI:** 10.3897/mycokeys.115.145774

**Published:** 2025-03-12

**Authors:** Jun Yan, Li Xiong, Li-Xun Yang, Zheng-Mi He, Ping Zhang, Ke Liao

**Affiliations:** 1 College of Life Science, Hunan Normal University, Changsha 410081, China Hunan Normal University Changsha China; 2 Hunan Provincial Demonstration Center of Forestry Seeding Breeding, Changsha 410329, China Hunan Provincial Demonstration Center of Forestry Seeding Breeding Changsha China; 3 Bureau of Forestry, Tongdao Dong Autonomous County, Huaihua 418500, China Bureau of Forestry, Tongdao Dong Autonomous County Huaihua China

**Keywords:** Clavariaceae, morphology, phylogeny, taxonomy

## Abstract

Based on morphological and molecular evidence, 12 specimens have been identified as belonging to three previously unrecognized species of *Clavaria*, which are here described as *C.divergens*, *C.orientalis*, and *C.tongdaoensis*. *Clavariadivergens* is characterized by its branched, white basidiomata. *Clavariaorientalis* and *C.tongdaoensis* are very similar to *C.zollingeri* in the field. However, *C.orientalis* is distinguished by its more robust branches, while *C.tongdaoensis* differs by its varied or paler color of basidiomata. A concatenated sequence dataset (ITS-nrLSU-*RPB*2) was used for multi-locus phylogenetic analysis. The phylogenetic tree of *Clavaria* showed that the three branched species each formed a distinct lineage with strong support. A key to the known branched species of *Clavaria* in China is provided.

## ﻿Introduction

Vaillant (1727) described three clavarioid species and first used the term “Clavaria” to name them. Subsequently, Linnaeus (1753) formally established the genus *Clavaria* Vaill. ex L. in *Species Plantarum* and described five species with branched basidiomata. However, as an increasing number of species with branches were discovered, infrageneric groups of *Clavaria* species with branched basidiomata, such as *Clavaria* α *Ramaria* Pers., Clavariatrib.Botryoideae Fr., and Clavariatrib.Ramariae Fr., were proposed (Persoon 1801; Fries 1821). With further research, species of *Clavaria* with branches have been sequentially segregated as separate genera, such as *Artomyces* Jülich, *Clavulina* J. Schröt., *Clavulinopsis* Overeem, *Ramaria* Fr. ex Bonord., and *Ramariopsis* (Donk) Corner (Corner 1950). At present, species of *Clavaria* with authentically branched fruit bodies are not common, with only seven species recognized before the present study (Léveillé 1846; Corner 1967; Lazo 1972; Furtado et al. 2016; Yan et al. 2020).

Among the seven branching species, *Clavariagriseolilacina* P. Zhang and *Clavariasinensis* P. Zhang are native to China and were described in 2020 (Yan et al. 2020). Their type specimens have been compared with the new species identified in the current study. In addition, *Clavariadiverticulata* A.N.M. Furtado & M.A. Neves and *Clavariamartinii* Corner are recorded only in the Americas, and their basidiomata are yellow (Corner 1967; Furtado et al. 2016). Therefore, the white or slightly yellowish pink *Clavariapumanquensis* Lazo and the cosmopolitan *Clavariazollingeri* Lév. (Corner 1950; Lazo 1972) are more directly relevant for careful differentiation from the three new species in this study.

In China, purple branching *Clavaria* species have often been identified as *C.zollingeri* in the past. However, a comparison of specimens collected during the past 20 years has revealed a clear morphological difference between species distributed in northern and southern China. In the present study, only specimens collected in Jilin Province (northern China) have larger basidiomata and longer branches and conform with *C.zollingeri*; specimens collected in Hubei and Hunan provinces (southern China) belong to species new to science based on morphological and molecular evidence. An additional new species, *C.divergens*, has a white, stably branched basidiomata, which is a very rare character combination in *Clavaria*.

## ﻿Materials and methods

### ﻿Morphological examination

Twelve specimens of the three new species were collected by the authors in Hunan or Hubei provinces between 2003 and 2022. Habitat photographs of basidiomata were taken in the field, and macromorphological data were recorded from fresh specimens. The color of the basidiomata was described with reference to color codes (Kornerup and Wanscher 1978) and color names (Ridgway 1912). Specimens were deposited in the Mycological Herbarium of Hunan Normal University (MHHNU), Changsha, China, after drying. Micromorphological features were recorded from microscopic observations. The handling of dried vouchers followed the procedures of Yan et al. (2023). The abbreviation [*n*/*m*/*p*] indicates that *n* basidiospores were measured from *m* basidiomata of *p* specimens. Dimensions of basidiospores are presented in the form (a–)b–c(–d), where a and d represent extreme values, and the range b–c comprises 90% of the measured values. All measurement data were analyzed with SPSS 14.0 (SPSS, Inc.). Q is the “length/width ratio” of a basidiospore in lateral view, and Q indicates the average Q of all basidiospores ± sample standard deviation.

### ﻿DNA extraction, PCR amplification, and sequencing

Total genomic DNA was extracted from dried vouchers using the modified cetyltrimethylammonium bromide method introduced by Doyle and Doyle (1987) or the Ezup Column Fungi Genomic DNA Purification Kit (Sangon Biotech, Shanghai, China). The primer pairs ITS4/ITS5 (White et al. 1990) and LR0R/LR5 (Vilgalys and Hester 1990) were used to amplify the internal transcribed spacer (ITS) region and the nuclear ribosomal large subunit (nrLSU) region, respectively. The primers f RPB2-5F, f RPB2-6F, and f RPB2-7.1R (Liu et al. 1999; Matheny et al. 2007) were used to amplify the RNA polymerase II second largest subunit (*RPB2*) region. The PCR reaction volume and thermal-cycling conditions followed those of Yan et al. (2022) and He et al. (2023). The PCR products were examined and sequenced by Sangon Biotech. Sequences generated in this study were deposited in GenBank.

### ﻿Alignment and phylogenetic analyses

The dataset used for phylogenetic analyses included the newly generated sequences and sequences downloaded from GenBank. Detailed information on the sequences is listed in Table 1.

**Table 1.** Voucher information and GenBank accession of taxa used in this study.

Identification	Specimen No.	GenBank No. (ITS)	GenBank No. (28S)	GenBank No. (RPB2)	Location
*Clavariaalboglobospor*a	JAC15834	OR567635	OR567767	—	New Zealand
*C.amonenoides*	Lueck4	KP965768	KP965786	—	Germany
*C.amonenoides*	MHHNU10306	ON228386	ON231688	ON246172	China
*C.amonenoides*	MHHNU10522	ON228387	ON231689	ON246173	China
*C.appendiculata*	AMB 18348	MN022549	MN018833	—	Italy
*C.apulica*	AMB 150	MT853065	MT853066	—	Italy
*C.argillacea*	K(M)126733	KC759438	JQ415931	—	United Kingdom
*C.argillacea*	BRACR 16025	KC759439	JQ415930	—	Slovakia
*C.aspersa*	MHHNU32157	ON228390	ON231692	ON246176	China
*C.aspersa*	MHHNU32397	ON228391	ON231693	ON246177	China
*C.asterospora*	BIO-Fungi 12390	KC759440	—	—	Spain
*C.atrofusca*	BRACR 13264	HQ606080	HQ606081	—	Norway
*C.atroumbrina*	K(M)143730	—	JN315789	—	United Kingdom
*C.calabrica*	ZT Myc 58697	MF972889	MF972885	—	Italy
*C.californica*	AMB 18558	MT055940	—	—	Italy
*C.californica*	TENN026785	HQ179660	—	—	USA
*C.citrinorubra*	TENN040464	HQ179661	HQ877686	—	Australia
*C.crosslandii*	BIO-Fungi 12762	KC759441	—	—	Spain
*C.cupreicolor*	TENN043696	KP257109	KP257187	—	New Zealand
***C.divergens***	**MHHNU8277**	**PQ819508**	**PQ814267**	—	**China**
***C.divergens***	**MHHNU9857**	**PQ819509**	**PQ814268**	**PO806984**	**China**
***C.divergens***	**MHHNU10164**	**PQ819510**	**PQ814269**	**PO806985**	**China**
***C.divergens***	**MHHNU10165**	**PQ819511**	**PQ814270**	**PO806986**	**China**
*C.echino-olivacea*	TENN043686	KP257110	KP257188	—	New Zealand
*C.flavipes*	BRACR 15121	KC759450	KC759472	—	Slovakia
*C.flavipes*	TENN063740	KP257119	EF535267	—	United Kingdom
*C.flavostellifera*	BIO-Fungi 10433	KC759461	JX069828	—	Slovakia
*C.flavostellifera*	BRACR 16695	KC759462	JX069827	—	Slovakia
*C.fragilis*	MHHNU10527	ON228394	ON231696	ON246179	China
*C.fragilis*	MHHNU32418	ON228395	ON231697	ON246180	China
*C.fragilis*	TENN033244	KP257121	KP257195	—	USA
*C.fumosa*	MR00170	JN214482	HQ877696	—	USA
*C.fumosa*	TENN060724	KP257126	KP257199	—	Russia
*C.fuscata*	JMB08181001	KP257128	HQ877691	KP257253	USA
*C.gibbsiae*	PDD 111979	OR567704	OR567794	—	New Zealand
*C.globospora*	TENN045945	KP257130	KP257201	—	USA
*C.greletii*	ERRO 2014102101	MF503244	—	—	Spain
*C.greletii*	C(F) s/n	—	JN416778	—	Denmark
*C.griseobrunnea*	BIO-Fungi 12566	KY091644	—	—	Spain
*C.griseolilacina*	MHHNU9722	MT028142	ON231725	ON246185	China
*C.griseolilacina*	MHHNU10149	MT028141	ON231726	ON246186	China
*C.hupingshanensis*	MHHNU7362	ON228396	ON231698	ON246181	China
*C.incarnata*	AMB 18345	MK908007	MK898930	—	Italy
*C.incarnata*	BIO-Fungi 12560	KC759452	—	—	Spain
*C.incarnata*	MA53113	KC759453	JQ415948	—	Spain
*C.lametina*	AMB 18933	OQ595227	OQ595225	OQ594954	Italy
C.cf.macounii	PK1536	KP257131	KP257202	KP257254	Canada
*C.megaspinosa*	JAC14897	OR567613	OR567751	—	New Zealand
*C.megaspinosa*	JAC16538	OR567650	OR567778	—	New Zealand
C.messapicaf.alborosea	AMB 18346	MN017594	MN017499	—	Italy
*C.messapica*	AMB 12800	—	KM486538	—	Italy
*C.messapica*	IHI-20Cla01	MW786738	MW786737	—	Germany
*C.musculospinosa*	PDD 82582	OR567692	OR567786	—	New Zealand
*C.neonigrita*	Ceska06112010	JN214481	JN214484	—	Canada
***C.orientalis***	**MHHNU6801**	**PQ819512**	**PQ814271**	—	**China**
***C.orientalis***	**MHHNU7352**	**PQ819513**	**PQ814272**	**PO806987**	**China**
***C.orientalis***	**MHHNU7586**	**PQ819514**	**PQ814273**	**PO806988**	**China**
***C.orientalis***	**MHHNU7767**	**PQ819515**	**PQ814274**	—	**China**
***C.orientalis***	**MHHNU32116**	**PQ819516**	**PQ814275**	**PO806989**	**China**
*C.parvispora*	BRACR 13266	—	MH727523	—	Norway
*C.parvispora*	BRACR 21309	—	MH727524	—	Slovakia
*C.pisana*	AMB 18620	MW355011	MW355012	—	Italy
*C.pseudoincarnata*	AMB 17377	MN017595	MN017500	—	Italy
*C.pseudoincarnata*	AMB 17379	MN017596	MN017501	—	Italy
*C.pullei*	MONI 2018122801	MW549781	MW549780	—	Spain
*C.pullei*	SAV F3139	KP257132	KP257203	KP257255	Czech Republic
*C.redoleoalii*	JAC14916	OR567617	OR567755	—	New Zealand
*C.redoleoalii*	JAC14917	OR567642	OR567772	—	New Zealand
*C.rosea*	TENN063100	KP257133	KP257205	KP257256	USA
*C.rosea*	TENN065117	KP257134	KP257206	KP257257	USA
*C.roseoviolacea*	JAC14915	OR567616	OR567754	—	New Zealand
*C.roseoviolacea*	JAC15786	OR567632	OR567764	—	New Zealand
*C.rubicundula*	JLH MyCoPortal 6603126	MK578690	—	—	USA
C.cf.rubicundula	JMB10061005	—	HQ877690	—	USA
*C.salentina*	AMB 010297	MF972892	MF972888	—	Italy
*C.sinensis*	MHHNU8198	MT028140	ON231727	ON246187	China
*C.sphagnicola*	BRACR 13593	KC759455	KC759471	—	Norway
*C.sphagnicola*	BRNM 747282	KC759456	KC759470	—	Czech Republic
*C.stegasauroides*	JAC14852	OR567586	OR567742	—	New Zealand
*C.stegasauroides*	PBM3373	—	HQ877698	KP257261	Australia
*C.stellifera*	IHI-19Cla01	OK239673	OK239677	—	Germany
*C.straminea*	BRACR 12807	KC759449	JQ415944	—	Slovakia
*C.subviolacea*	JAC14150	OR567566	OR567726	—	New Zealand
*C.tenuipes*	ARAN-Fungi 11295	MW248489	MW248513	—	Spain
***C.tongdaoensis***	**MHHNU11091**	**PQ819517**	**PQ814276**	**PO806990**	**China**
***C.tongdaoensis***	**MHHNU11093**	**PQ819518**	**PQ814277**	**PO806991**	**China**
***C.tongdaoensis***	**MHHNU11094**	**PQ819519**	**PQ814278**	**PO806992**	**China**
*C.tyrrhenica*	ZT Myc 58698	MF972890	MF972886	—	Italy
*C.ypsilonidia*	PDD 46673	NR174884	NG079629	—	New Zealand
*C.ypsilonidia*	TENN042411	KP257140	KP257210	KP257262	New Zealand
*C.zollingeri*	MHHNU10528	ON228397	ON231699	ON246182	China
*C.zollingeri*	MHHNU10548	ON228398	ON231700	ON246183	China
*C.zollingeri*	MHHNU10550	ON228399	ON231701	ON246184	China
*C.zollingeri*	TENN064095	KP257141	HQ877700	KP257263	USA
*C.zollingeri*	TENN58652	AY854071	AY639882	AY480940	—
*Mucronellaflava*	IO.16.84	MT232354	MT232307	—	Sweden
*Mucronella* sp	PDD 95742	HQ533013	—	—	New Zealand

The ITS, nrLSU, and *RPB*2 sequences were respectively aligned using MAFFT v7.471 (Katoh and Standley 2016) and manually edited in BIOEDIT v7.2.5 (Hall 1999) where necessary. The combined matrix of ITS, nrLSU, and *RPB*2 sequences was assembled with SEQUENCEMATRIX 1.7.8 (Vaidya et al. 2011). The concatenated sequence dataset was analyzed using maximum likelihood (ML) and Bayesian inference approaches with RAXML v8.0.20 (Stamatakis 2006) and MRBAYES v3.2.7 (Ronquist and Huelsenbeck 2003), respectively. The ML analysis was conducted using the GTR+Gamma evolutionary model with 1000 bootstrap replicates. The Bayesian inference analysis ran for 1,000,000 generations using the GTR+I+G optimal evolutionary model selected with MRMODELTEST v2.4 (Nylander 2004). The phylogenetic trees were visualized using FigTree v1.4.2 (Rambaut 2012) and further refined using Adobe Photoshop CS6 and Illustrator CS5 (Adobe Systems, Inc., San Jose, CA, USA).

## ﻿Results

### ﻿Phylogenetic analyses

**Figure 1.**
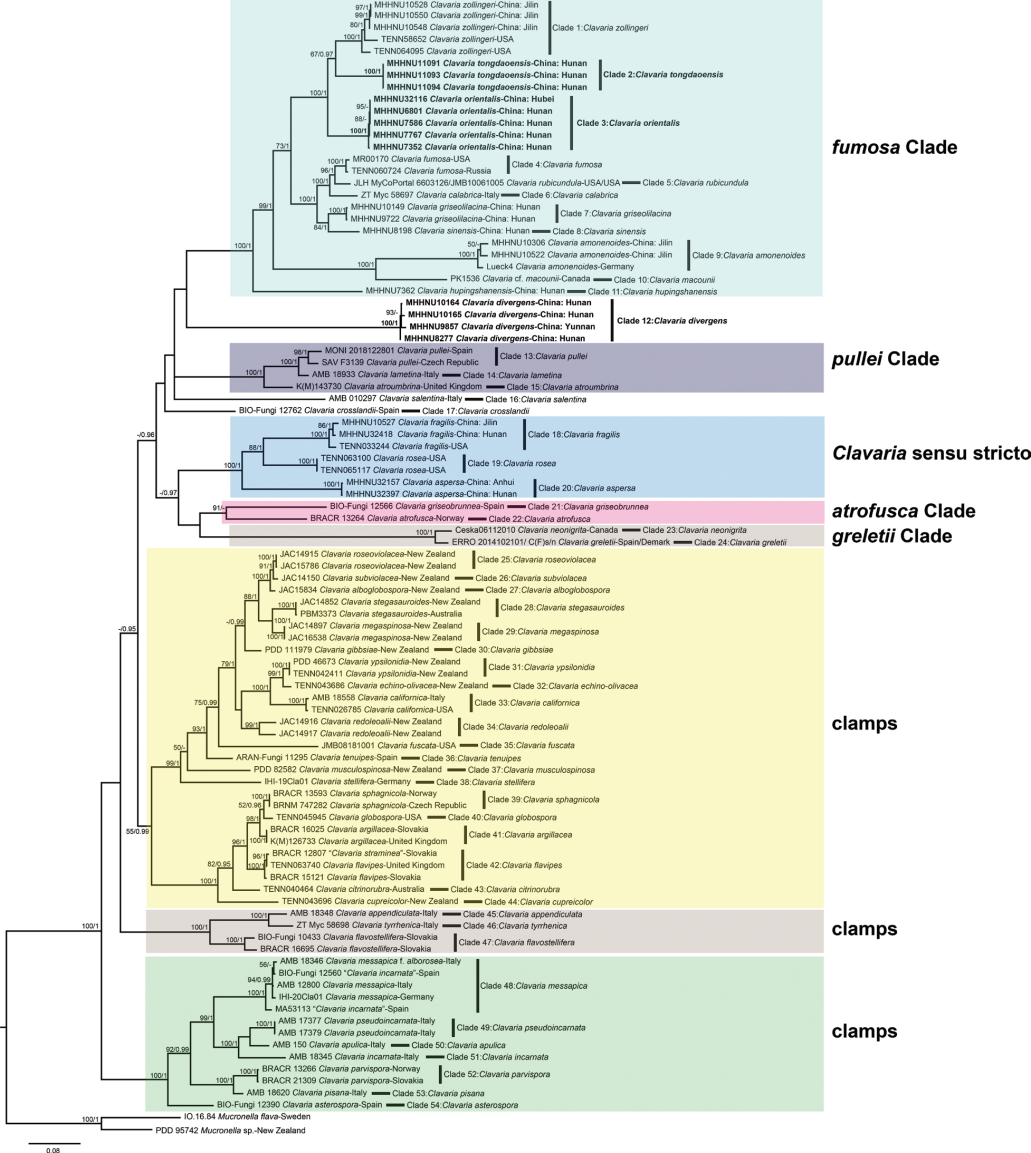
Phylogenetic relationships of *Clavaria* species inferred from ITS, nrLSU, and *RPB*2 sequences under the maximum likelihood optimality criterion. Bayesian posterior probabilities over 0.95 and bootstrap values over 50% are reported at nodes (BI/MP); the sign “–” means under the reported level. Our new species are shown in boldface text.

The data matrix consisted of 210 sequences (90 ITS, 88 nrLSU, and 32 *RPB*2) from 97 samples, among which 33 (12 ITS, 12 nrLSU, and 9 *RPB*2) were newly generated in the present study. The aligned concatenated ITS–nrLSU–*RPB*2 dataset, comprising a total of 2450 nucleotide positions, was used for the BI and ML analyses. The ML analysis yielded a tree topology with branch lengths and support values represented in Fig. 1, and the BI analysis yielded an almost identical phylogenetic construction (not shown). Bayesian posterior probabilities > 0.95 and bootstrap values > 50% are shown at the nodes in Fig. 1.

The ML and Bayesian analyses showed that two accessions of *Mucronella* Fr. (as the outgroup) and 54 species of *Clavaria* formed independent lineages, named Clade 1 to Clade 54 in turn. Eight main clades were resolved among the 54 species of *Clavaria*, which is similar to previous studies (Kautmanová et al. 2012; Birkebak et al. 2016). Clades 1 to 11 formed a well-supported (ML 100%/BI 1) clade (*Clavariafumosa* clade); the *Clavariapullei* clade (ML 100%/BI 1) included *Clavariaatroumbrina*, *Clavarialametina*, and *Clavariapullei*; *Clavaria* sensu stricto (ML 100%/BI 1) included the species *Clavariaaspersa*, *Clavariafragilis* (the type for the genus), and *Clavariarosea*; *Clavariaatrofusca* and *Clavariagriseobrunnea* formed the *Clavariaatrofusca* clade (ML 91%); and *Clavariagreletii* and *Clavarianeonignta* formed the *Clavariagreletii* clade (ML 100%/BI 1). The remaining species were resolved in three main clades; these species are united in possessing a loop-like clamp at the base of the basidium. Our three new species each formed a distinct monophyletic lineage with strong support (ML 100%/BI 1). The new species *Clavariadivergens* formed a distinct lineage (Clade 12) sister to the *Clavariafumosa* clade, to which *Clavariacrosslandii* (Clade 17) and *Clavariasalentina* (Clade 16) were also phylogenetically close. The other two new species, *Clavariaorientalis* and *Clavariatongdaoensis*, formed genetically distinct lineages (Clade 2 and Clade 3) that were phylogenetically closest to *Clavariazollingeri* within the *Clavariafumosa* clade.

### ﻿Taxonomy

#### ﻿ Clavaria divergens

Taxon classificationFungiAgaricalesClavariaceae

P. Zhang & Ju. Yan sp. nov.

4CF53AAB-C5B0-5883-9D03-8E08D9AD2063

857600

Figs 2 , 3

##### Etymology.

*divergens* (Latin) refers to the basidioma with dichotomous to irregularly divergent branches.

##### Holotype.

China • Yunnan Province: Malipo County, Donggan Town, alt. 1580 m, 23°21'41.98"N, 105°09'44.17" E, 6 August 2018, P. Zhang (MHHNU9857).

##### Diagnosis.

This species differs from other species within Clavariasubg.Syncoryne in its white branched basidiomata and 4-spored basidia.

##### Description.

**Figure 2.**
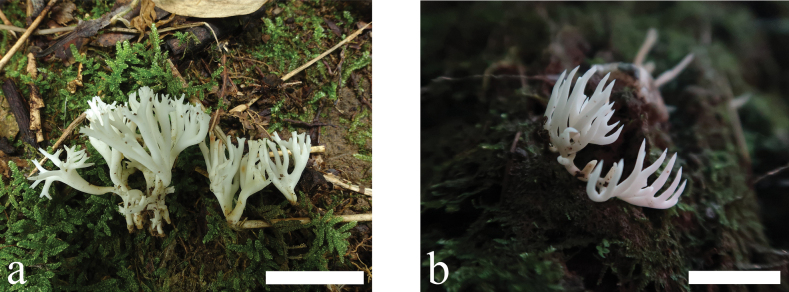
Basidiomata of *Clavariadivergens***a** MHHNU9857 **b** MHHNU10165. Scale bars: 2 cm.

Basidiomata (Fig. 2a, b) branched, brittle, scattered, or gregarious clusters; clusters 10–50 mm high, 10–30 mm broad; branches terete, 1–3 mm wide, 2–4 times, dichotomous, or irregularly divergent in the ultimate rank; branch tips subacuminate, often antler-like or claw-like. Fertile part coralloid, smooth, slightly curved, occasionally with a longitudinal depression in center, white [A1; White]. Apex white, becoming yellowish or tawny with age. Sterile part distinct, white, smooth, without tomentum and mycelial patch. Flesh concolorous with surface of basidiomata.

**Figure 3.**
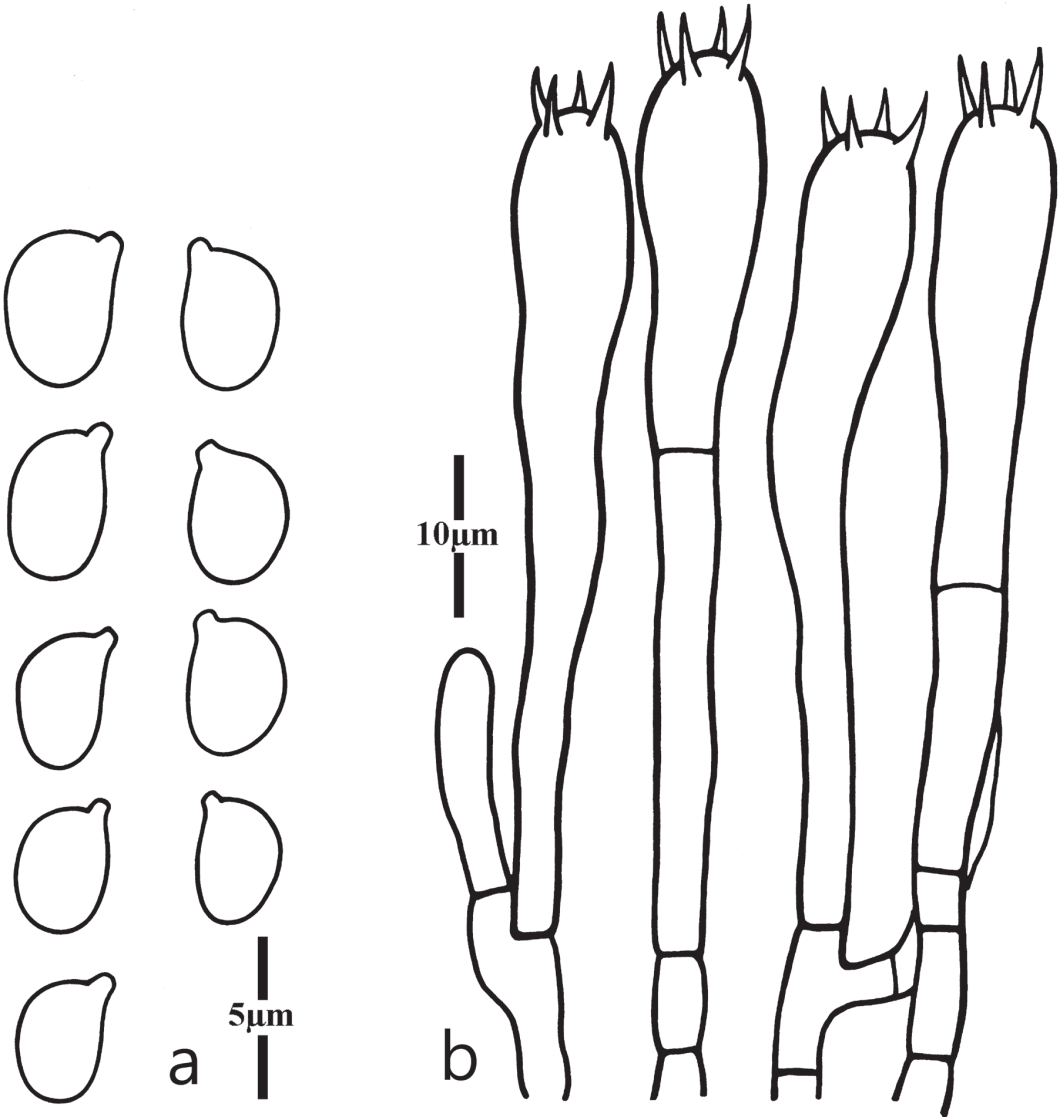
Microscopic features of *Clavariadivergens* (MHHNU9857) **a** basidiospores **b** basidia.

Basidiospores (Fig. 3a) [100/6/4] (4.0)4.2–5.0 × (2.4)2.7–3.8(4.0) μm [Q = (1.25)1.26–1.56(1.60), Q = 1.39 ± 0.10], mostly ellipsoid, sometimes also broadly ellipsoid, smooth, hyaline, nonamyloid, thin-walled; hilar appendix small (<1.0 μm in length). Basidia (Fig. 3b) 48–65 × 6.5–9.0 μm, clavate, 4-spored, hyaline, thin-walled or slightly thick-walled, sometimes with secondarily septated; sterigmata up to 5.2 μm long. Incrustations or crystals absent. Hyphae of the context parallel, thin-walled, hyaline, cylindrical to inflated, secondarily septated. Clamp connections absent in all parts of basidiomata.

##### Habitat, ecology, and distribution.

Scattered or gregarious in humus layers of soil under mixed coniferous–broadleaved forests or broadleaved forests. Basidiomata produced in summer, usually throughout the months of July to August; known from subtropical zones of Central and Southwestern China.

##### Additional specimens examined.

China • Hunan Province: Yongshun County, Xiaoxi National Nature Reserve, alt. 1068 m, 28°47'45.84"N, 110°12'13.89"E, 28 August 2014, P. Zhang (MHHNU8277); • Guzhang County, Gaofeng Town, alt. 573 m, 28°40'45.42"N, 110°08'28.56"E, 23 July 2020, Ju. Yan (MHHNU10164, MHHNU10165).

#### ﻿ Clavaria orientalis

Taxon classificationFungiAgaricalesClavariaceae

P. Zhang & Ju. Yan sp. nov.

03C72319-8183-55D9-8A84-7886304EEAF3

857601

Figs 4 , 5

##### Etymology.

*orientalis* (Latin), meaning eastern, refers to the occurrence of the species in East Asia.

##### Holotype.

China • Hunan Province: Shimen County, Hupingshan Nature Reserve, alt. 1828 m, 30°02'58.50"N, 110°31'24.90"E, 11 September 2012, P. Zhang (MHHNU7767).

##### Diagnosis.

Differs from *Clavariazollingeri* in its stout branches, lesser degree of branching, and shorter basidia.

##### Description.

**Figure 4.**
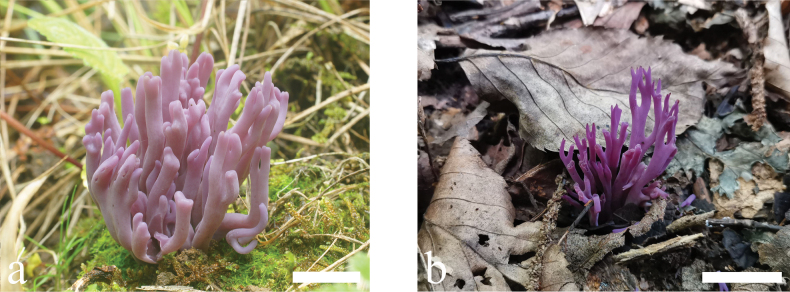
Basidiomata of *Clavariaorientalis***a** MHHNU7767 **b** MHHNU32116. Scale bars: 2 cm.

Basidiomata (Fig. 4a, b) branched, brittle, gregarious to caespitose clusters; clusters 50–80 mm high, 10–30 mm broad; branches terete, 1–3 mm wide, 1–4 times, dichotomous; branch tips obtuse, broadly rounded, or narrowly rounded. Fertile part coralloid, smooth, obviously curved or slightly twisted, deep amethyst [15A4-7, 15B4-6, 16A4-6; Amparo Purple, Lobelia Violet, Vinaceous Purple] to lilac [14A2-3, 15A2-3; Pale Vinaceous Purple, Pale Lobelia Violet], and changing to pale greyish purple [16A2, 17A2; Lavender Gray, Pale Payne’s Gray, Pale Verbena Violet] with age. Apex concolorous with lower part, becoming yellowish or tawny with age. Stipe distinct, sterile, smooth, often terete, semi-translucent, hygrophanous, and darker than the fertile part, sometimes flattened and pallid. Flesh concolorous or slightly paler than surface of basidiomata.

**Figure 5.**
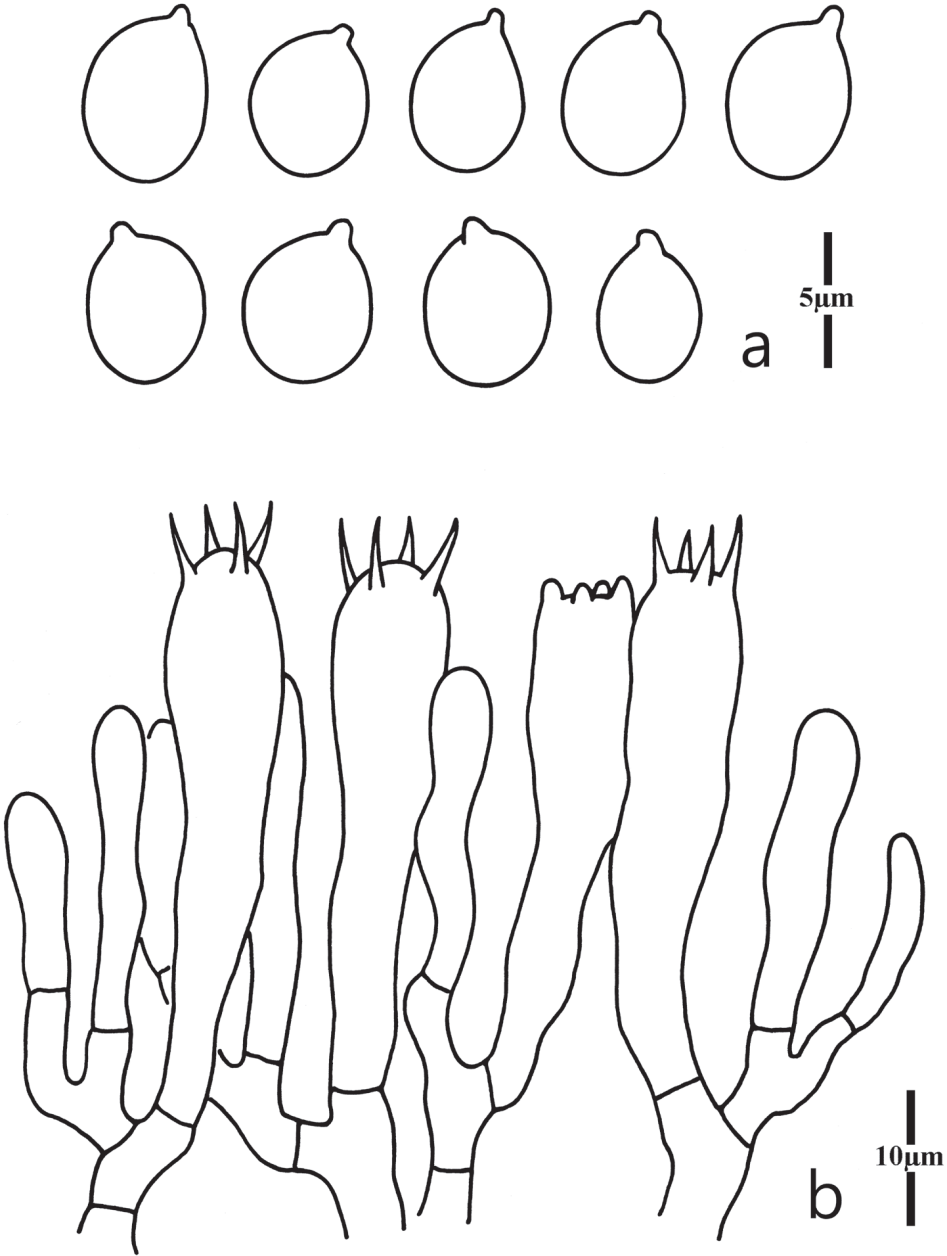
Microscopic features of *Clavariaorientalis* (MHHNU7767) **a** basidiospores **b** basidia.

Basidiospores (Fig. 5a) [100/5/5] (4.8)5.0–6.0 × 4.0–5.0(5.5) μm [Q = (1.09)1.13–1.38(1.43), Q = 1.21 ± 0.11], mostly broadly ellipsoid, sometimes ellipsoid or subglobose, smooth, hyaline, nonamyloid, thin-walled; hilar appendage present (<2.0 μm in length). Basidia (Fig. 5b) 34–48 × 5.0–8.0 μm, clavate, 4-spored, hyaline, thin-walled; sterigmata below 5.0 μm long. Incrustations or crystals absent. Hyphae of the context parallel, thin-walled, hyaline, cylindrical to inflated, secondarily septated. Clamp connections absent in all parts of basidiomata.

##### Habitat, ecology, and distribution.

Gregarious to caespitose in humus layers of soil under broadleaved forests, coniferous forests, or mixed coniferous–broadleaved forests. Basidiomata produced in summer or autumn, usually throughout the months of July to September; known from subtropical zones of southern China.

##### Additional specimens examined.

China • Hunan Province: Sangzhi County, Badagongshan National Nature Reserve, alt. 1500 m, 29°46'58.17"N, 110°4'51.68"E, 22 July 2003, P. Zhang (MHHNU6801); • Shimen County, Hupingshan Nature Reserve, alt. 1828 m, 30°02'58.50"N, 110°31'24.90"E, 31 August 2010, P. Zhang (MHHNU7352); • 26 September 2011, P. Zhang (MHHNU7586). • Hubei Province: Hefeng County, Mulinzi National Nature Reserve, alt. 1413 m, 30°03'32.17"N, 110°12'34.35"E, 1 August 2020, Z.H. Chen (MHHNU32116).

#### ﻿ Clavaria tongdaoensis

Taxon classificationFungiAgaricalesClavariaceae

P. Zhang & Ju. Yan sp. nov.

BAD57780-344C-55DB-9250-906F289B2192

857602

Figs 6 , 7

##### Etymology.

*tongdaoensis* (Latin) refers to the type locality in Tongdao County, Hunan Province, China.

##### Holotype.

China • Hunan Province: Tongdao County, Fengshuwan Forest Park, alt. 400 m, 26°09'45.66"N, 109°46'31.52"E, 6 July 2022, P. Zhang and Li-Xun Yang (MHHNU11094).

##### Diagnosis.

Distinguished from *Clavariaorientalis* and *Clavariazollingeri* by its smaller basidiomata and basidiospores.

##### Description.

**Figure 6.**
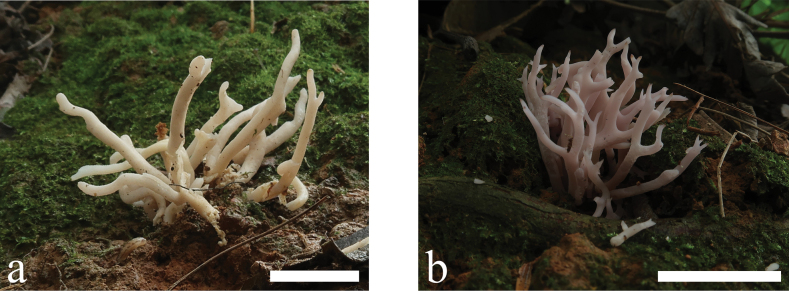
Basidiomata of *Clavariatongdaoensis***a** MHHNU11093 **b** MHHNU11094. Scale bars: 2 cm.

Basidiomata (Fig. 6a, b) branched, brittle, gregarious to caespitose clusters; clusters 25–45 mm high, 30–40 mm broad; branches terete, 2–3 mm wide, 1–3 times, dichotomous; branch tips narrowly rounded or awl-shaped. Fertile part coralloid, smooth, often curved or slightly twisted, pale purple to pale purplish pink [13A2-3, 14A2-3; Pale Lavender Violet, Pale Lobelia Violet, Pale Purplish Vinaceous], turning white with age. Apex concolorous with lower part, becoming yellowish or tawny with age. Stipe distinct, sterile, smooth, terete, semi-translucent, hygrophanous, slightly darker color than the fertile part. Flesh concolorous with surface of basidiomata.

**Figure 7.**
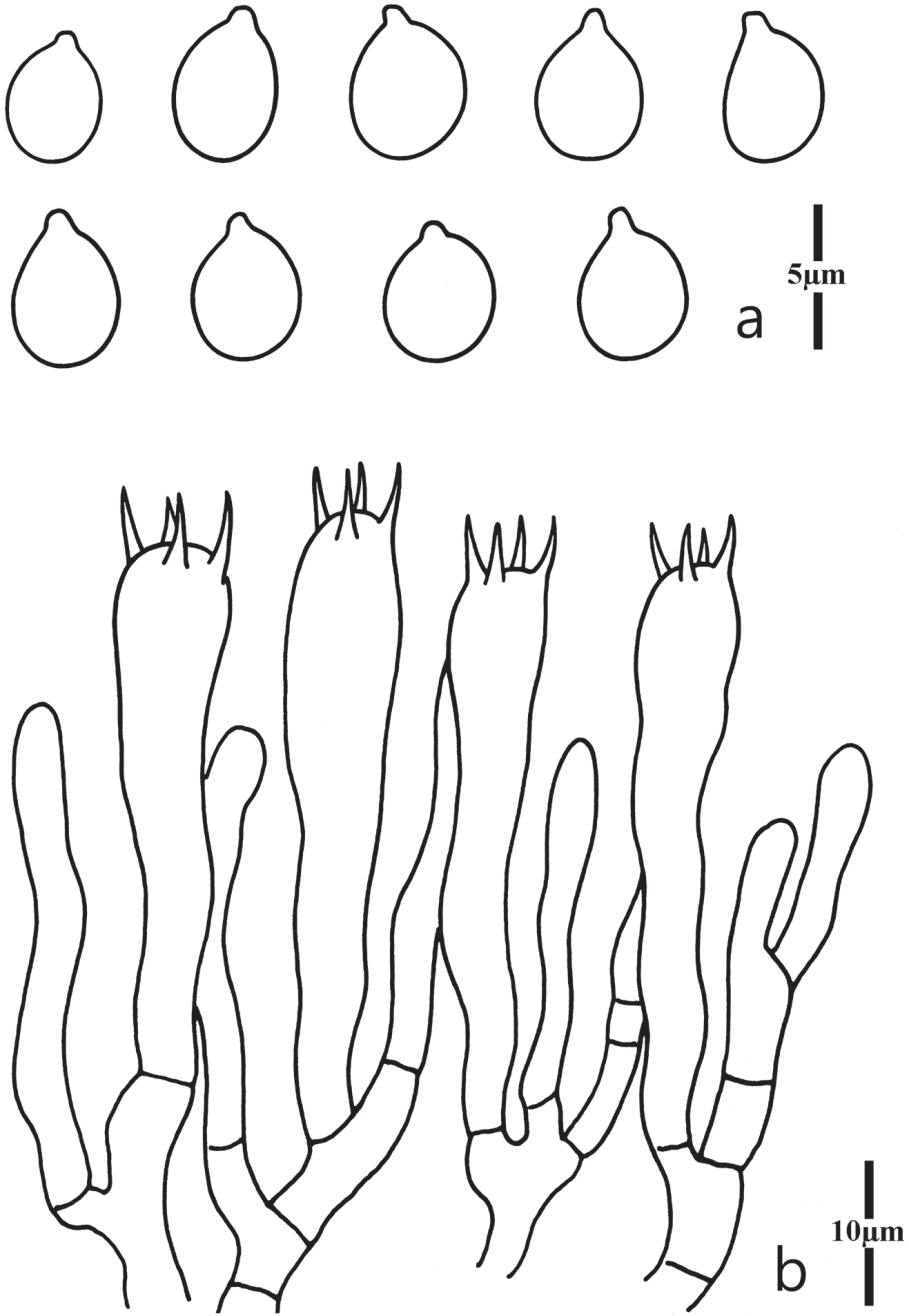
Microscopic features of *Clavariatongdaoensis* (MHHNU11094) **a** basidiospores **b** basidia.

Basidiospores (Fig. 7a) [100/5/3] 3.5–5.0 × 3.0–4.2(4.5) μm [Q = (1.05)1.06–1.33, Q = 1.16 ± 0.08], broadly ellipsoid, sometimes subglobose, smooth, hyaline, nonamyloid, thin-walled; hilar appendage present. Basidia (Fig. 7b) 26–43 × 6.5–8.0 μm, clavate, 4-spored, hyaline, thin-walled; sterigmata below 5.0 μm long. Incrustations or crystals absent. Hyphae of the context parallel, thin-walled, hyaline, cylindrical to inflated, secondarily septated. Clamp connections absent in all parts of basidiomata.

##### Habitat, ecology, and distribution.

Gregarious to caespitose in humus layers of soil under broadleaved forests. Basidiomata produced in summer; known only from the type locality, China.

##### Additional specimens examined.

China • Hunan Province: Tongdao County, Fengshuwan Forest Park, alt. 400 m, 26°09'45.66"N, 109°46'31.52"E, 6 July 2022, P. Zhang and Li-Xun Yang (MHHNU11091, MHHNU11093).

## ﻿Discussion

In this study, three new species of Clavariawithinsubg.Syncoryne were identified from specimens collected in China. The three species have in common the absence of a loop-like clamp connection at the base of the basidia and obvious branching of the basidiomata. Before the present study, only seven species within *Clavaria* were known to stably produce branched basidiomata, namely, *C.diverticulata* A.N.M. Furtado & M.A. Neves (Furtado et al. 2016), *C.griseolilacina* P. Zhang (Yan et al. 2020), *C.hupingshanensis* P. Zhang & Ju. Yan (Yan et al. 2022), *C.martinii* Corner (Corner 1967), *C.pumanquensis* Lazo (Lazo 1972), *C.sinensis* P. Zhang (Yan et al. 2020), and *C.zollingeri* Lév. (Léveillé 1846). Among these seven branched species, *C.zollingeri* is of greatest relevance in the present study.

**Figure 8.**
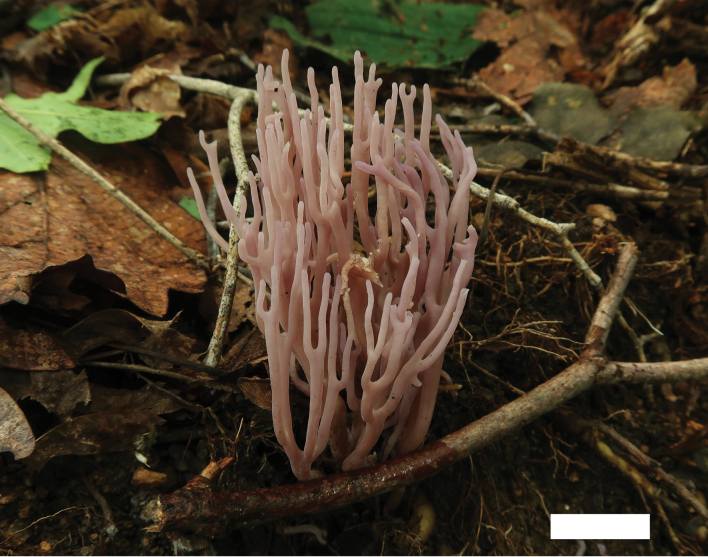
Basidiomata of *Clavariazollingeri* (MHHNU10528). Scale bars: 2 cm.

We initially mistook *C.orientalis* and *C.tongdaoensis* to be *C.zollingeri* based on the purple color of their basidiomata until we collected material of *C.zollingeri* (MHHNU10528, Fig. 8) in Jilin Province (northern China) that matched a previous description of that species. A comparison of the specimens revealed the differences among the three species. More specifically, Corner noted that, for *C.zollingeri*, the basidioma is 15–75 mm high, the spores are 4.0–7.0 × 3.0–5.0 μm, the basidia are 50–60 × 7–10 μm, and the sterigmata are 4–7 μm (Corner 1950). Franchi and Marchetii (2021) noted that the basidiomata of *C.zollingeri* are up to 80 mm high and the basidia are 50–60 × 8–10 μm. In contrast, *C.orientalis* discovered in southern China has shorter basidia (34–48 × 5.0–8.0 μm) and shorter sterigmata (< 5.0 μm long) than those of *C.zollingeri*. An additional species collected from southern China, *C.tongdaoensis*, has shorter basidia (26–43 × 6.5–8.0 μm), smaller spores (3.5–5.0 × 3.0–4.2(4.5) μm), and shorter sterigmata than *C.zollingeri*. In addition, the branches of *C.orientalis* often are not as profuse as the branches of *C.zollingeri*, and, compared with *C.zollingeri*, the basidiomata of *C.tongdaoensis* are smaller (25–45 mm high). *Clavariadivergens* is quite unique within *Clavaria*. Based on the color of the basidiomata, *C.divergens* is similar to *C.fragilis* or *C.gibbsiae*, but *C.fragilis* and *C.gibbsiae* always have a simple basidiomata, occasionally once-furcate. Compared with the branched *Clavaria* species mentioned above, *C.divergens* is distinctive with its white basidiomata and subacuminate branch tips.

In the phylogeny for *Clavaria*, 54 species are supported based on molecular data, which is much higher than previous records or predictions. For example, 28 species of *Clavaria* were recognized in Ainsworth and Bisby’s “Dictionary of the Fungi”, 10^th^ edn. (Kirk et al. 2008), and Olariaga et al. (2015) estimated that the genus comprises 30–35 species. Two of the new species, *C.orientalis* and *C.tongdaoensis*, together with *C.zollingeri*, formed a clade with strong support (ML 100%/BI 1), a relationship consistent with the morphological similarity of the three species. In the phylogenetic analysis, sequence data for five specimens of *C.zollingeri* were included, two of which were collected in the United States and the other three were collected from Jilin Province, China. This finding supports the contention that *C.zollingeri* in North America and populations in northern China are conspecific. In contrast, eight specimens collected from southern China were genetically distinct from *C.zollingeri* and showed morphological differences and thus were identified as new species, named here *C.orientalis* and *C.tongdaoensis*. Moreover, *C.divergens* was indicated to be genetically very distinct in the phylogenetic tree. Although the phylogeny indicated that *C.divergens* has a close relationship with the *Clavariafumosa* clade and represented a sister lineage to that clade, the node was not statistically supported. Nevertheless, the phylogenetic analysis supported its genetic distinctness and monophyly as an independent lineage and verified its identity as a previously unrecognized species. However, its phylogenetic relationships with other species of *Clavaria* require further research.

In summary, most species of *Clavaria* are unbranched, but three branching species are described in this article. Among them, *C.orientalis* and *C.tongdaoensis* are distinguished from *C.zollingeri*, which is considered to be distributed only in northern China. *Clavariadivergens* is the first species discovered in China with stable white branches. The records of these three species enrich the species diversity of the genus *Clavaria* and increase the number of species with branched basidiomata in the genus.

### ﻿Key to branched species of *Clavaria* in China

**Table:** 

1	Basidiomata white to pink	**2**
–	Basidiomata purple	**3**
2	Basidiomata 10–50 mm tall, white	***C.divergens***
–	Basidiomata 35–70 mm tall, rose-white to seashell-pink	***C.hupingshanensis***
3	Basidiomata sparsely branched	***C.griseolilacina***
–	Basidiomata profusely branched	**4**
4	Fruiting body usually lighter colored	**5**
–	Fruiting body color usually darker colored	**6**
5	Basidiomata 30–70 mm tall, basidiospores 5.0–6.0 × 3.5–4.5 μm	***C.sinensis***
–	Basidiomata 25–45 mm tall, basidiospores 3.5–5.0 × 3.0–4.2 μm	***C.tongdaoensis***
6	Basidiomata branching stout, 1–4 times, distribution in southern China	***C.orientalis***
–	Basidiomata branching slim, 3–5 times, distribution in northern China	***C.zollingeri***

## Supplementary Material

XML Treatment for Clavaria divergens

XML Treatment for Clavaria orientalis

XML Treatment for Clavaria tongdaoensis

## ﻿Additional information

### Author ORCIDs

Jun Yan https://orcid.org/0000-0002-2832-8046

Li Xiong https://orcid.org/0009-0007-3568-7737

Li-Xun Yang https://orcid.org/0009-0003-3086-4424

Zheng-Mi He https://orcid.org/0000-0001-8754-3427

Ping Zhang https://orcid.org/0000-0002-8751-704X

### Data availability

All of the data that support the findings of this study are available in the main text or Supplementary Information.

## Supplementary materials

Supplementary material 1 ITS sequence dataset Data type fas Explanation note The ITS sequences were respectively aligned using MAFFT v7.471, and manually edited in BIOEDIT v7.2.5 where necessary. https://binary.pensoft.net/file/1278631This dataset is made available under the Open Database License (http://opendatacommons.org/licenses/odbl/1.0/). The Open Database License (ODbL) is a license agreement intended to allow users to freely share, modify, and use this Dataset while maintaining this same freedom for others, provided that the original source and author(s) are credited.Author: Jun Yan

Supplementary material 2 LSU sequence dataset Data type fas Explanation note The LSU sequences were respectively aligned using MAFFT v7.471, and manually edited in BIOEDIT v7.2.5 where necessary. https://binary.pensoft.net/file/1278632This dataset is made available under the Open Database License (http://opendatacommons.org/licenses/odbl/1.0/). The Open Database License (ODbL) is a license agreement intended to allow users to freely share, modify, and use this Dataset while maintaining this same freedom for others, provided that the original source and author(s) are credited.Author: Jun Yan

Supplementary material 3 *RPB2* sequence dataset Data type fas Explanation note The *RPB2* sequences were respectively aligned using MAFFT v7.471, and manually edited in BIOEDIT v7.2.5 where necessary. https://binary.pensoft.net/file/1278633This dataset is made available under the Open Database License (http://opendatacommons.org/licenses/odbl/1.0/). The Open Database License (ODbL) is a license agreement intended to allow users to freely share, modify, and use this Dataset while maintaining this same freedom for others, provided that the original source and author(s) are credited.Author: Jun Yan
